# *De Novo* Transcriptome Assembly and Comparative Analysis of Differentially Expressed Genes in *Prunus dulcis* Mill. in Response to Freezing Stress

**DOI:** 10.1371/journal.pone.0104541

**Published:** 2014-08-14

**Authors:** Sadegh Mousavi, Arghavan Alisoltani, Behrouz Shiran, Hossein Fallahi, Esameil Ebrahimie, Ali Imani, Saadollah Houshmand

**Affiliations:** 1 Department of Plant Breeding and Biotechnology, Faculty of Agriculture, Shahrekord University, Shahrekord, Iran; 2 Institute of Biotechnology, Shahrekord University, Shahrekord, Iran; 3 Department of Biology, School of Sciences, Razi University, Bagh-e-Abrisham Kermanshah, Iran; 4 Department of Crop Production and Plant Breeding, Faculty of Agriculture, Shiraz University, Shiraz, Iran; 5 School of Molecular and Biomedical Science, The University of Adelaide, Adelaide, Australia; 6 Department of Horticulture, Seed and Plant Improvement Institute (SPII), Karaj, Iran; University of North Carolina at Charlotte, United States of America

## Abstract

Almond (*Prunus dulcis* Mill.), one of the most important nut crops, requires chilling during winter to develop fruiting buds. However, early spring chilling and late spring frost may damage the reproductive tissues leading to reduction in the rate of productivity. Despite the importance of transcriptional changes and regulation, little is known about the almond’s transcriptome under the cold stress conditions. In the current reserch, we used RNA-seq technique to study the response of the reporuductive tissues of almond (anther and ovary) to frost stress. RNA sequencing resulted in more than 20 million reads from anther and ovary tissues of almond, individually. About 40,000 contigs were assembled and annotated *de* *novo* in each tissue. Profile of gene expression in ovary showed significant alterations in 5,112 genes, whereas in anther 6,926 genes were affected by freezing stress. Around two thousands of these genes were common altered genes in both ovary and anther libraries. Gene ontology indicated the involvement of differentially expressed (DE) genes, responding to freezing stress, in metabolic and cellular processes. qRT-PCR analysis verified the expression pattern of eight genes randomley selected from the DE genes. In conclusion, the almond gene index assembled in this study and the reported DE genes can provide great insights on responses of almond and other *Prunus* species to abiotic stresses. The obtained results from current research would add to the limited available information on almond and Rosaceae. Besides, the findings would be very useful for comparative studies as the number of DE genes reported here is much higher than that of any previous reports in this plant.

## Introduction

Drought, high salinity, and low temperatures are among major abiotic factors affecting plants’ geographical distribution and productivity. Due to drastic changes in climate, exposure of plants to these stresses is expected to increase in the near future. Hence, it is important to study the mechanisms by which plants would overcome such naturally occurring stresses.

In particular, cold stress, including chilling (<20°C) and frost (<0°C) temperatures, causes tissue injuries, delay in growth and reduction of photosynthesis. Plants respond to low temperatures by altering the expression of thousands of genes, thereby changing cellular, physiological, and biochemical processes [1], [2], [3]. Thus, analysis of gene expression would be a valuable tool to understand the dynamics of transcriptome and possibly the manipulation of the gene expression pattern of plants.

Following the rapid progress in sequencing technologies, whole RNA sequencing have been used for expression profiling and its potential in transcriptome studies has been proven. The exhaustive profile of transcriptome provided by RNA-seq approach allows absolute rather than relative gene expression measurements, detection of novel transcribed regions and gene isoforms. RNA-seq has been successfully applied to quantify RNA levels under both abiotic and biotic stresses in different model species including *Arabidopsis taliana* [4], rice [5], maize [6], peach [7] and many other species.

The Rosaceae family ranks third in economic importance of the plant families in temperate regions [8]; it includes many species such as almond, apple, apricot, blackberry, cherry, peach, raspberry, rose and strawberry, valued for nuts, fruits and flowers. High throughput sequencing was also appropriately conducted to the genome and transcriptome sequencing as well as genome wide expression analysis of Rosaceae including apricot [9], apple [10], peach [11], [12] and sweet cherry [13]. However, some members of this family such as almond are less studied using the new sequencing technologies.

Almond (*Prunus dulcis* Mill.), a perennial plant, is an important fruit tree in many areas [14], [15], [16]. But, spring frost injury is a major limiting factor in the production of almond [17], [18], [19]. Since almond is an early fruit tree, blooming in late winter or early spring [17], it is exposed to late-spring frost, which could result in reduction or even total lose of the yield [15], [17]. The spring frost could damage trees from the early blooming stage to anthesis [20], [21]. Imani et al. [21] reported that the temperature at which flower buds are injured depends on their stage of development. The authors discussed that almond buds are more resistant during the winter, whereas during the blooming (popcorn and anthesis) stage they are less tolerant to frost. In addition, there was genetic diversity for cold resistance among genotypes and varieties of almond due to structural, physiological, phenological and morphological features of genotypes [22].

Different studies evaluated the frost injury in almond trees, however, molecular processes involved in the cold response are poorly investigated in this plant [23]. Although, Barros et al. [23] recently demonstrated the role of *PdCBF2* in cold acclimation of almond, to our current knowledge, there is no study on the molecular responses of almond to frost stress.

Additionally, despite the importance of transcriptome analysis and high potential of RNA-seq technique, surprisingly little is known about the transcriptome of almond. A qucik search of NCBI for *Prunus dulcis* Mill. resulted in only 4,485 ESTs. Furthermore, there is yet little information about transcriptional changes and their regulation in almond and other *Prunus* species under abiotic stresses, and in particular, frost injury.

Here, using RNA-seq approach, we have carried out the first global analysis of almond’s transcriptome (in the reproductive tissues) under control and freezing conditions.

## Results and Discussion

### Overview of RNA-Seq Data

**Figure 1:**
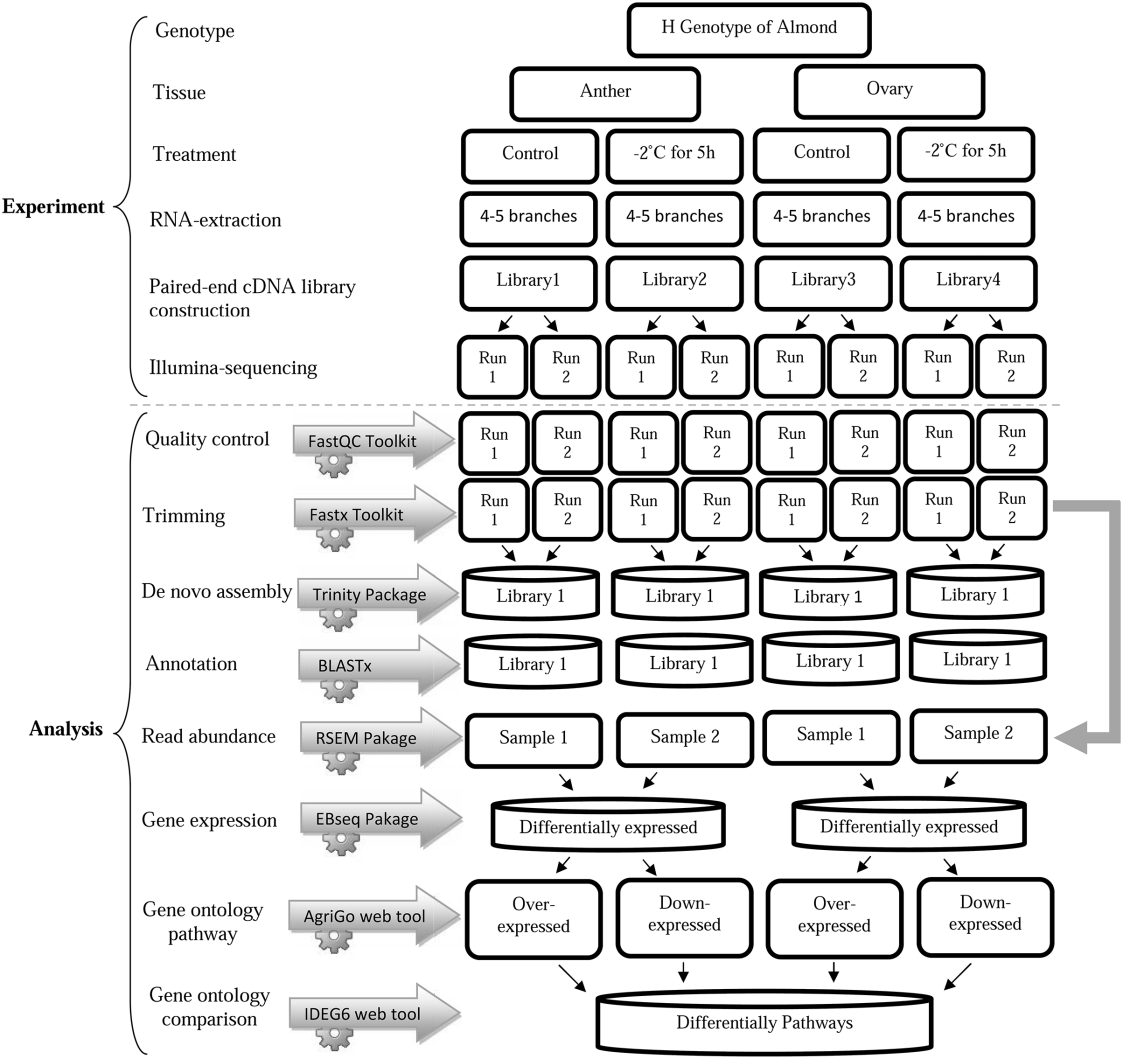
Our pipeline for RNA-seq analysis in almond under control and frost stress conditions. Two almond tissues (anther and ovary) were subjected to untreated and −2°C conditions. Following the RNA exteraction, cDNA library was constructed and sequencing was performed using an IIlumina platform. *De novo* transcript assembly was conducted after quality control and trimming the reads and then contigs were annotated. Transcript aboundance and differentially expressed genes were analyzed using RSEM and EBseq, respectively. Finally, gene ontology were determined for each set of genes in the DE list.

In the current study, four RNA samples of almond (H genotype), including control ovary (HCO), freezing-stressed ovary (HSO), control anther (HCA) and freezing-stressed anther (HSA), were sequenced and analyzed. The workflow of the study is presented in Figure 1. Pair-ended runs, 4.7 to 6.9 million reads, with an average quality of 38 and an average length of 101 nucleotides, were resulted for ovary and anther (Table 1). The GC contents of the reads were 45% for HSA and HSO samples and 46% for the HCA and HCO samples. The amounts of GC contents were approximately similar to the previously reported value for peach transcriptome (44%) [7], and it was a bit higher compared to that of sweet cherry (42%) [13]. The highest number of reads was obtained for HSA (6,588,049 reads), and the least was yielded by HCA (4,676,340 reads) (Table 1). The higher amounts of reads could increase the coverage efficiency and result in better estimation of gene expression. Nevertheless, different amount of reads has been sequensed in plants, which is mostly depended on sequencing platforms as well as cDNA preparation. Socquet-Juglard et al. [7] sequenced about fifty million reads in peach using illumina genome analyzer. Chen et al. [12] recently reported only 1.5 million sequenced reads for two libraries of peach using 454 S-FLX platform.

**Table 1:** Basic statistics of RNA-seq reads in almond obtained from Illumina HiSeq-2000.

Sample	Total Reads	Read length (nt)	GC contents	Average Quality score%
**HSA**	6588049	101	45%	38
**HCA**	4676340	101	46%	38
**HSO**	6223556	101	45%	38
**HCO**	6963246	101	46%	38

The qualtiy of reads in Fastq files were analyzed using FastQC software. The reads were subjected to vigorous quality filtering using FastX toolkit before further analysis.

Abbreviations: HSA, stressed anther of H genotype; HCA, control anther of H genotype; HSO, stressed ovary of H genotype; HCO, control ovary of H genotype.

### *De Novo* Transcriptome Assembly and Annotation of The *De Novo* Contigs

Following quality control (QC) and trimming the reads, the remaining reads were used for construction of transcriptome, using Trinity assembly package. A summary of the assembly statistics is presented in Table 2. For HSA sample, we have successfully assembled 44,477 contigs with minimum length of 200 and maximum length of 11,888 nucleotides, which was the longest contigs among all the assemblies. HCA sample contained the lowest number of contigs (produced 38,920), where maximum size of contigs was 7,145 nucleotides. We have also calculated the N50 as a measure of assemblies’ quality [24]. The highest N50 was 1,558 for HSO sample and HCA represented the lowest N50 of 1,204. This results were in agreement with other published transcriptomes in plants, where *de* *novo* assembley from Illumina reads were conducted for *Brassica rapa* (1482) and *Zea mays* (1612) [24]. High values of N50 in our data can indicate the effectiveness of assemblies. The variation in N50 may be due to differences between tissues and/or treatments, which can also be observed in the previous studies [13], [24].

**Table 2:** Statistics of *de* *novo* transcriptome assembly and annotation.

Sample	Num. ofcontigs	Min. length(bp)	Max. length(bp)	N50	Annotation byBLASTX (%)	Reads mapped topeach genome (%)	Reads mapped back tocontigs (%)
HSA	44477	200	11888	1484	94.54	80.73	79.42
HSO	41283	200	6888	1558	93.16	79.75	87.23
HCA	38920	200	7145	1204	91.63	81.26	82.39
HCO	48562	200	8256	1444	93.49	79.22	85.69

*De novo* assembley was performed using Trinity package following the standard manual. Annotation was achieved using BLASTX program. Mapping statistics of reads to the transcriptome assemblies and peach genome are also provided, which was obtained using Bowtie v2 package.

Abbreviations: HSA, stressed anther of H genotype; HCA, control anther of H genotype; HSO, stressed ovary of H genotype; HCO, control ovary of H genotype.

Annotations of the assemblies were performed using BLASTX implemented in the Bioportal server located at the Oslo University. More than 91% of all the contigs were successfully annotated (Table 2). We have obtained 94.54%, 93.16%, 91.63% and 93.49% annotations for HAS, HSO, HCA and HCO assemblies, respectively.

**Figure 2:**
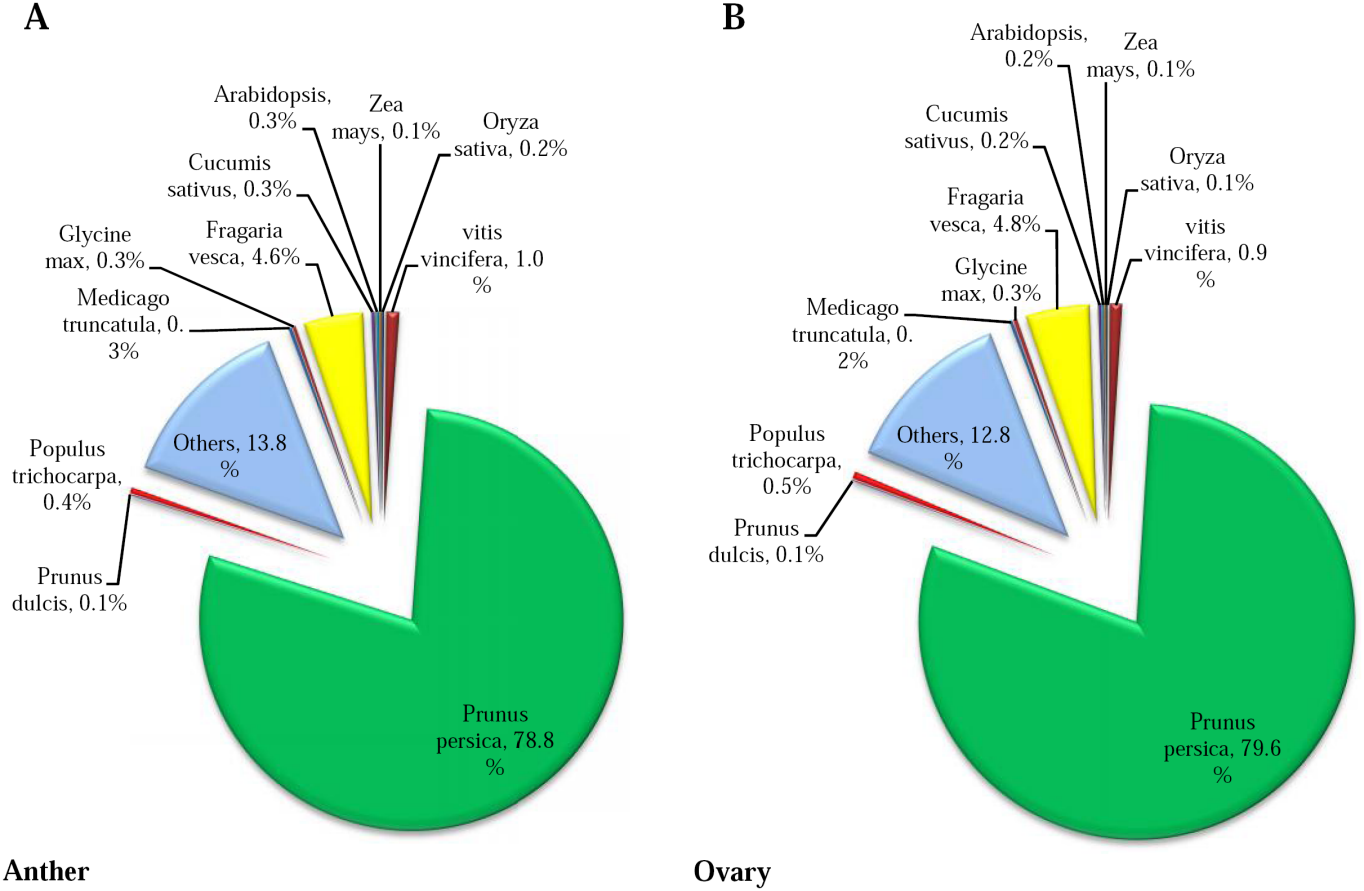
Distribution of differentially expressed genes of almond under frost stress. Venn diagram indicating the number of differentially expressed contigs under frost in anther and ovary tissues of almond.

The distribution of the best hits are presented in Figure 2. The maximum homology was found with *Prunus persica* (78.8% and 79.6% in anther and ovary, respectively) followed by *Fragaria vesca* and *Vitis vicifera*. The remaining contigs showed similarity with a wide range of species (to a very low extent in each of the species). The high level of annotation similarity with *Prunus persica* was in agreement with Scalabrin et al.’s study, where they have reported 73.7% coverage of almond reads with peach genome [25]. These similarities could indicate that the quality of our assembley is good enough to proceed to the next step of analysis.

### Quality Validation of *De Novo* Contigs

To further assess the quality of the assemblies, the original reads were mapped back to the constructed assemblies (Table 2). Almost 80% of the reads were successfully mapped back to their assemblies, with HSO and HSA showed the highest and the lowest mapping percentages, respectively. These percentages of the mapped reads are much higher than the ratio previously reported in rice (60%) [26], peach (73.8%–76.9%) [7] and sweet cherry (66%) [13], whereas it was lower in comparison with the study of Wang et al. [27] on peach (90%).

We have also mapped our reads to the *Prunus persica* v1.0 genome [11]. Almost 80% of the reads could be properly mapped to the peach reference genome. Anther samples (HCA and HSA) showed the mapping percentages of 81.26% and 80.73%, respectively. In contrast, ovary samples (HSO and HCO) showed 79.75% and 79.22% mapping coverage, respectively (Table 2). These results were in consistent with our findings in the annotation step, where a large number of contigs were well matched with peach genes. This could show high accuracy of our contigs assembly and could reflect the presence of high similarity between almond and peach genomes. The high level of homology between peach and almond as well as other *Prunus* genomes has been also reported in our own and other previous reports [28], [29], [30].

### Gene Expression Profiling and identification of Differentially Expressed Genes

Gene expression profile for each samples were extracted using RSEM package (version -1.2.6) and Bowtie v.1 for mapping reads. The gene expression patterns for each sample are presented in Tables S1 and S2 in File S1, in which gene expressions are given as transcripts per million (TPM). Gene expression profiles of 36,491 and 38,459 genes were obtained from anther and ovary samples, respectively. We have also investigated the expression profiles for those genes shared by both ovary and anther tissues (Table S3 in File S1). This list contains 13,279 common genes with detectable expression level in both tissues.

**Figure 3:**
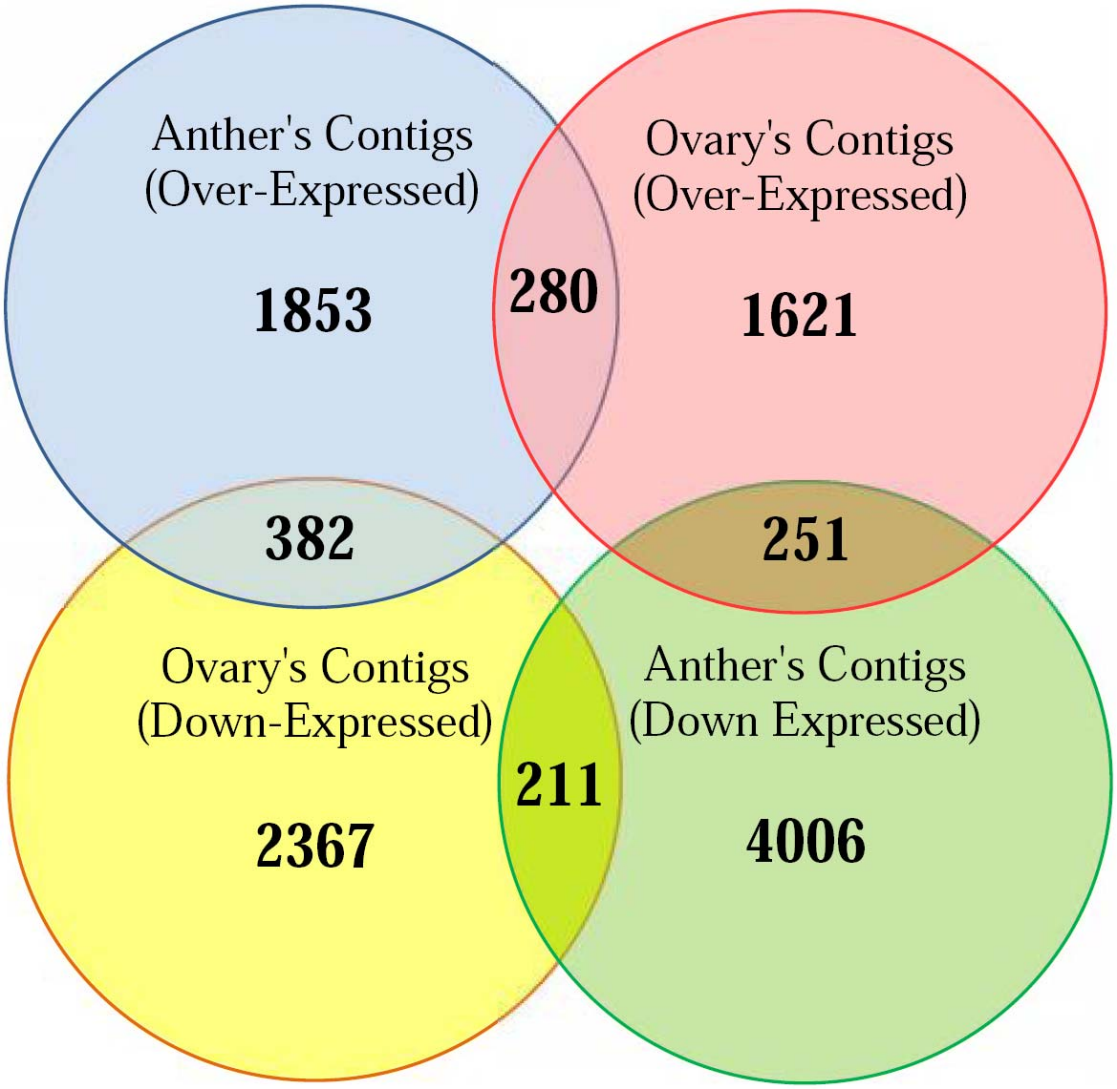
Homology of almond transcriptome to other plant species. The percent of BLASTX top-hit with different plant species. Maximum homology of almond transcripts were observed with peach (*Prunus persica*).

Differentially expressed genes were identified using EBSeq package. The fold change cut-off was set at two fold with *p*-value<0.05 (Tables S4 and S5 in File S1). The log_2_ of real fold changes and its *p*-value are presented for 6,926 and 5,112 genes, obtained by comparing stressed and control samples collected from anther and ovary, respectively. Abundance of ubiquitously expressed genes are varied across different tissues. For instance, approximately 55–67% of genes in *Arabidopsis* and 60–70% in human and mouse are expressed in tissue-specific patterns [12]. Transcriptomic studies have revealed that about 68% of genes are expressed in whole flower tissues of peach [27]. Of the 2,415 over-expressed genes in anther, 280 and 382 genes shared similar patterns to those expressed in control and stressed ovary samples, respectively. While, among 4,455 down-expressed genes of anther, 251 and 211 were showed similarity with genes in control and stressed ovary samples, respectively (Figure 3). Altogether, 5,859 and 3,988 genes were shown DE specifically in anther or ovary.

The applied RSEM pakage estimates the expression of the representative alternative splicing (AS) isoforms in Trinity assemblies. Alternative splicing is common in plants and over 20% of plant genes produce two or more transcript isoforms [30]. In peach, 838 AS events have been discovered through EST sequencing (The International Peach Genome Initiative 2013). Intrestingly, results showed different AS events under freezing stress. As an example, chaperone protein DnaJ-like isoform 2 was highly over-expressed under freezing stress in anther, whereas no significant DE was detected for isoform 1 (Table S4 in File S1). However, the reference genome is needed to identify the accurate ratio and the type of AS events in almond. Furthermore, more cDNA libraries sequencing are required, for different tissues, developmental stages and a range of stress conditions, to get full view of AS events in almond.

**Figure 4:**
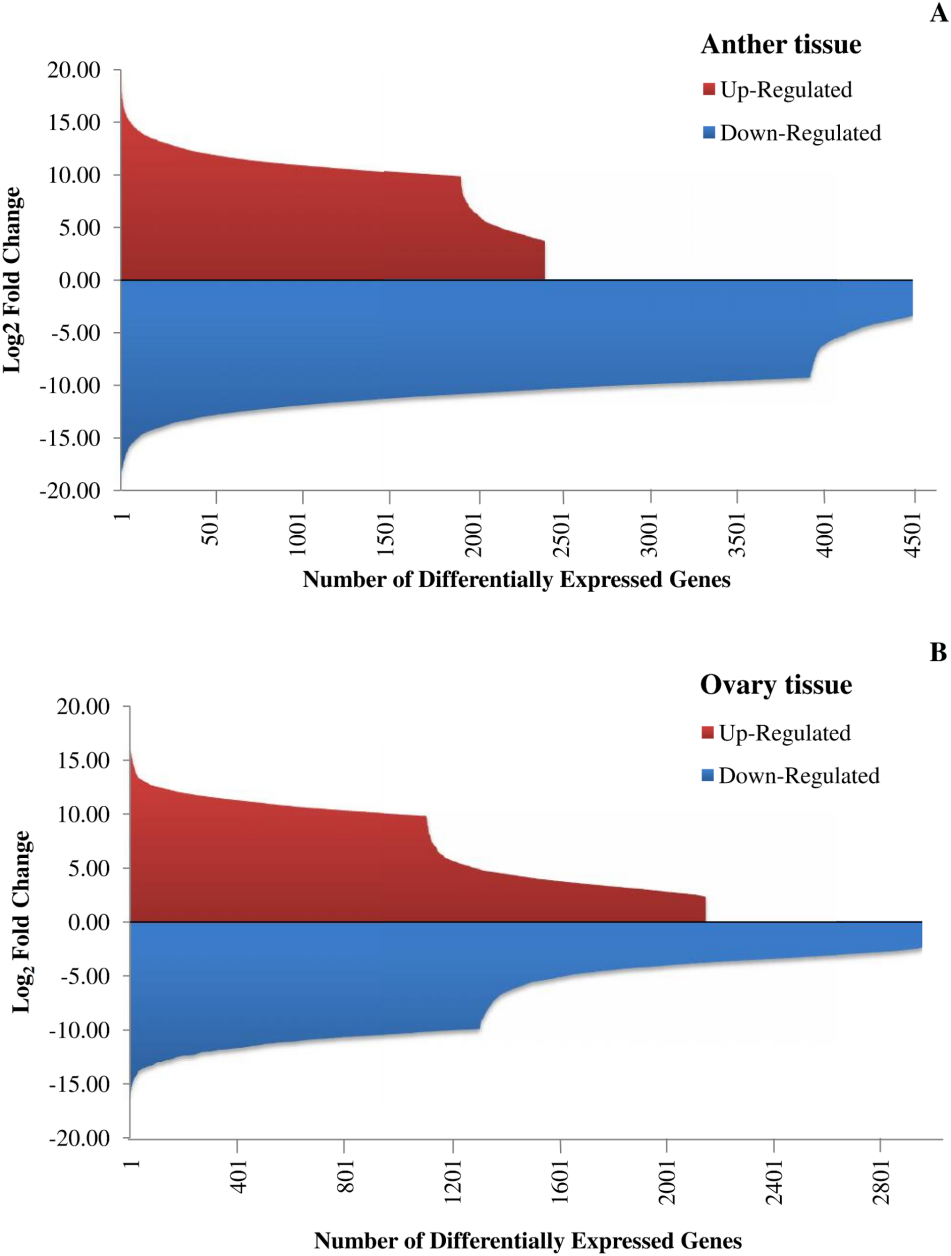
Distribution of log_2_ fold change for differentially expressed genes. A and B are distribution of log_2_ FC in anther and ovary, respectively.

The distributions of the DE genes were further analyzed using edgeR package. Volcano graph for both sets of experiments (HSA *vs.* HCA and HSO *vs.* HCO) highlighted normal distribution of gene expression (data not shown). We have also assesed distribution of log_2_ fold changes for over- and down-expressed genes in each tissue (Figure 4). While in anther about 66% of the DE genes illustrated log_2_ FC higher than 10 fold, almost 43% of total DE genes were in ovary tissue.

As additional evaluation of gene expression results, some of the house keeping genes were selected according to Socquet-Juglard et al [7]. We did not detect a significant differentially expressed level for tubulin, catalase or GAPDH, all of which expressed with a DE below our threshold (Data not shown).

**Figure 5:**
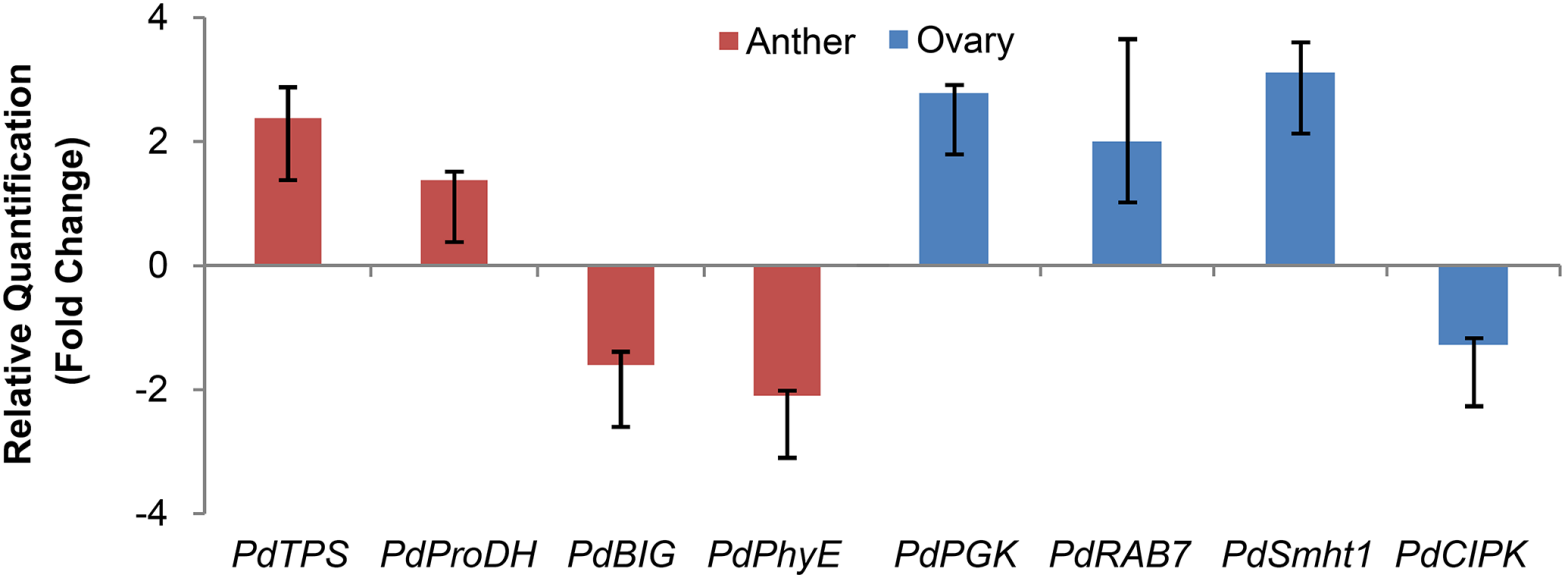
qRT-PCR analysis of almond genes under frost stress. The transcription level for each gene was calculated by quantitative RT-PCR and normalized against *PdActin*. Data represent means ± standard deviation (n = 2).

Moreover, using a random sets of genes in the DE genes list, we validated the RNA-seq expression profiling by qRT-PCR technology. The accession number and putative name of the selected gene are listed in Table 3. Statistically, *PdProDH* (EMJ16473) and *PdTPS* (EMJ15164) were significantly over-expressed in anther, with *p-value*<0.05, whereas the expression level of *PdBIG* (EMJ04259) and *PdPhyE* (EMJ15756) statistically decreased in anther under frost (Figure 5). In the case of ovary tissue, *PdPGK* (EMJ16473), *PdRab7* (EMJ03050) and *PdShmt1* (EMJ26905) were recorded as over-expressed genes (*p-value*<0.05) (Figure 5). On the contrary, *PdCIPK* (EMJ08660) was significantly down-expressed under frost condition.

**Table 3:** The list of primers used for gene expression analysis by quantitative Real Time PCR.

First Hit Annotation(Gene Accession No.)	Putative Gene Name	Primer Sequences 5′–>3′	Annealing (°C)	Amplicon (bp)
AM491134	Pd_Act1	F: TCCTGAAGAGCACCCAGTTC	59.31	116
		R: TGGCAACATACATAGCAGGC	58.32	
EMJ04259	Pd_BIG	F: CCTTCTTACTGCTGTGGCATC	58.99	126
		R: GCCTTTGACGTTGCTTGGTT	59.9	
EMJ15164	Pd_TPS	F: ACTACCTTGCCCCTGAACTC	59.02	169
		R: TAAATCACCCCTCGTCTTCCG	59.52	
EMJ16473	Pd_ProDh	F: CAGCAGCAGTCTTTCCTTTGC	60.34	164
		R: AAGCCAATGTCCCCTTATCCG	60.13	
EMJ15756	Pd_PhyE	F: AAGCTGCCCGTTTCTTGTTC	59.33	132
		R: GTGACCGGAGGGTTGAATTG	58.83	
EMJ03050	Pd_Rab7	F: TGGCACGAGGAGTTTCTCAAG	60.27	135
		R: GCACCAGTCCTTTGCTTTCTTC	60.29	
EMJ15113	Pd_PGK	F: TCCAATCCCATCCACCCATC	59.15	189
		R: CTCAGTCGGTTCGTCTCTGG	59.83	
EMJ26905	Pd_Shmt1	F: GGGTGTCAATGTCCAGCCTT	60.25	157
		R: TGCAGAGACCTTCTTCCCAC	59.31	
EMJ08660	Pd_CIPK	F: AGAGGCTATGATGGGGCAAC	59.52	159
		R: ACCAAAAGAGAGCCAAGGGG	59.89	

Corresponding annealing temperatures and amplicon sizes are shown for each gene.

Abbreviations: F, Forward primer; R, Reverse primer.

Interstingley, some of the above outlined genes have been previously evaluated in a variety of abiotic stresses, including serine hydroxymethyltransferase (*Shmt1*) [31], proline dehydrogenase (*ProDH*) [32] and trehalose phosphate synthase (*TPS*) [33], [34]. For instance, Kohl et al. reported that drought stress induced the activity of the enzymes of proline metabolism such as *ProDH* in bacteroids, suggesting that proline may be imported to the symbiosomes as a substrate for bacteroids during periods of stress [32]. Besides, proline oxidation, mediated by *ProDH*, could serve as an alternative for oxidative phosphorylation to produce ATP, NADPH and phosphorylated sugars needed for anabolic pathways [35].

The similar expression trend of these genes in both analytical techniques (Figure 5, Table S4 and S5 in File S1) demonstrates the quality of our gene expression profiling and its usage for further analysis. However, because RNA-seq and qRT-PCR experiments were conducted on different samples collected in two different years (2013 for RNA-seq analysis and 2014 for qRT-PCR), there are notable differences between the exact level of gene expression values obtained by these two methods. Nevertheless, in general, the trends of gene expression obtained from qRT-PCR were in good agreement with that of RNA-seq expression profiling.

### Gene Ontology Pathways

**Figure 6:**
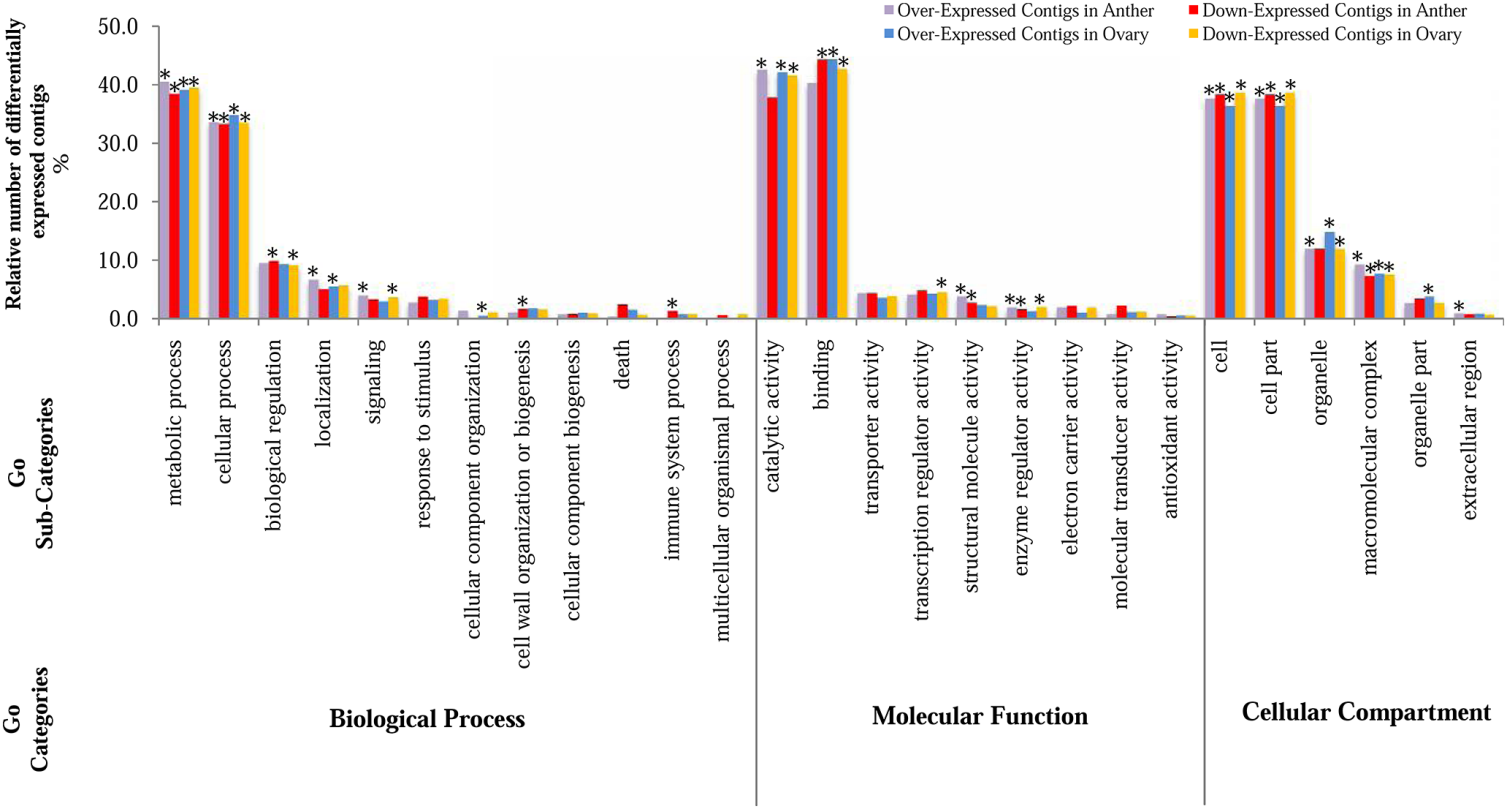
Gene ontology classes. Percentage of differentially expressed contigs annotated for Biological Process, Cellular Component and Molecular Function GO categories. *indicates a significant estimation at 0.05.

Functional classifications of differentially expressed genes were achieved using a gene ontology (GO) analysis tool via AgriGO website [36]. The DE genes were assigned into three classes of GO; biological processes, molecular functions and cellular compartments (Tables S6 to S8 in File S1). Comparison of GO list using IDEG6 [37] showed significant differences of GO terms between DE lists as well as tissues (data not shown). To investigate the most represented GO terms under frost stress, a comparative study was conducted between the DE lists and peach reference genome deposited in the AgriGo database (Tables S9–S12 in File S1). Figure 6 illustrates distinct distribution of the main GO categories and sub- categories in the differentially expressed genes. Most of the DE genes were classified in metabolic processe (39% in both tissues) and cellular process (33% in both tissues) followed by other classes with a wide range of sub-classes.

The transcriptional changes of genes involved in metabolic and cellular processes were previously demonstrated under different environmental conditions and developmental stages [7], [13], [38], [39]. For instance, Dang et al. found that the GO categories, including oxidoreductase activity, catalytic activity and response to stress were significantly enriched among DE genes compared with the whole transcriptome background. Interestingly, small GTPase mediated signal transduction, intracellular signaling cascade and cellular carbohydrate metabolic process as well as many different processes were over-represented in anther. On the contrary, protein modification and signaling processes were recorded as highly significant altered processes in ovary. This is in agreement with a whole-genome-wide transcriptional analysis where environmental/stress responses were manifested in post-transcriptional events, often leading to changes in RNA stability [40].

Gene ontology annotations also represented diverse functional activities corresponding with the mentioned biological processes. The highest numbers of DE genes were categorized in the catalytic and binding activities, which accounted for more than 40% in both tissues. These results were in agreement with earlier studies, which reported the over-representation of the mentioned terms under variety of stresses [6], [7], [38], [39], [41], [42], [43]. Among different functional activities, regulation of signal transduction was significantly enriched among DE genes compared with the peach transcriptome in both tissues. In contrast with previous studies, which highlighted the role of ABA related events under stress [38], no such events were detected in our study.

The significant over-representation of the biological pathways and functional activities suggests the importance of DE genes in response to cold stress. Nonetheless, it did not mean that such processes are restricted to cold stress responses of almond since most of them were reported to be involved in cold stress response in other plants [42], [44].

The significant differences were also observed for the cellular compartment sub-classes (Figure 6). A knowledge of the cellular compartments is essential for understanding the metabolism process and behavior of organells. In our study, we found significant representation of cell membrane protein components in both anther and ovary of almond (Tables S9–S12 in File S1). Membrane systems, which are known to be the primary site of frost injury in plants, suffer multiple forms of damage caused by frost-induced cellular dehydration [45]. Chilling stress usually causes injury such as the ROS formation in plant cells, which seriously damages membrane lipids, proteins and nucleic acids and disturbs the homeostasis of the organism [46]. Plants can improve cold tolerance by increasing the concentrations of unsaturated fatty acids and phospholipids [47]. Interestingly, we identified a number of over-expressed genes in anther and ovary that are involved in lipid metabolic processes (Tables S9 and S11 in File S1). The significant presence of lipid metabolic process only in the over-expressed list in both tissues allows us to hypothesize that over-expression of these genes may be related to cold tolerance. The results were in agreement with previous studies [48], [49]. For example, a higher unsaturated/saturated fatty acid ratio was showed to be correlated with cold acclimation in heat-treated pomegranate fruit [48].

We also searched for the protein domain of DE genes in mentioned significant GO terms (data not shown). AP2-domain and protein kinase were over-represented among different identified domains. A variety of protein domains are important for the specificity of interactions between signaling proteins. Furthermore, conserved regions known as “docking domains” or “kinase interaction motifs” (KIMs), govern the interactions of phosphatases, MAPK kinases, scaffolds and substrates with specific MAPKs [50]. Previously, it was reported that different protein kinase families are activated by osmotic stresses [51]. These proteins are important components in the signal transduction pathways of various environmental signals in plants [51], [52]. Interestingly, GO analysis showed the significant categories of protein kinase activity for DE genes in both tissues.

The AP2 domain is found in many plant genes and is related to the DNA binding region of ethylene response element binding proteins, which are known to be involved in ethylene signal transduction [53]. The AP2 domain is composed of a three-strand β-sheet and an α-helix, with amino acids 14 and 19 of the β-sheet being important for the binding to the target DNA [54], [55]. This domain is considered to recognize the C-repeat/DRE, cis-acting DNA regulatory element that stimulates transcription in response to low temperature and water deficit core sequences [56]. Interestingly, CBF/DREB1 was highly over-expressed (log_2_ FC = 11.19) under freezing in ovary, while no significant alternation was observed in anther (Table S4 and S5 in File S1). Low-temperature signaling pathways mediated by the C-repeat binding factor (CBF)/DREB1 family of transcription factors (TFs) are primary regulatory candidates in this process. Furthermore, Barros et al. [23] showed a progressive increase in transcript abundance of *PdCBF2* (*Prunus dulcis* C-repeat binding factor) during autumn in response to cold acclimation.

Additionally, more detailed analysis is underway in our group to shed light on different aspects of gene expression pattern of almond’s response to frost. The results of such study would be presented elsewhere.

In conclusion, we have conducted the first highthrouput analysis on gene expression pattern of almond’s reproductive tissues under frost stress. In addition to providing more than 40,000 valuable transcript seuqnces to public database, which was the result of our *de* *novo* assembley, we found that more than 7,000 genes showed signifigant alteration in their expression pattern. Gene ontology analysis revealed a number of important biological processes, molecular functions and cellular comportments in response to frost. The identified substantial number of novel transcripts could vastly improve the genome annotation of almond and other Rosaceae members. Furthermore, the results could be used to develop informative markers for breeding programs of almond. Moreover, our results will be also very helpful for the future of functional genomic research in almond and other fruit trees. This study also provides great clues to the whole transcriptional changes under low temperatures. However, further researches are needed to examine the usages of detected genes as biomarkers for marker-assisted breeding of almond or candidates for gene transfer in order to produce cold resistant plants.

## Methods

### Plant Materials and Stress Condition

**Figure 7:**
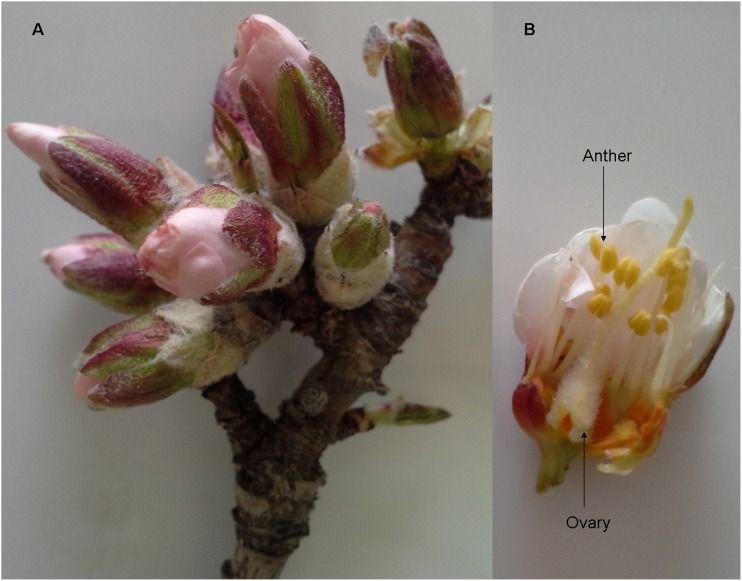
A side view of almond flowers used in this study. The popcorn stage of almond flower showed in A. The position of anther and ovary tissues in almond which were collected for the RNA-seq analysis showed in B.

To conduct the transcriptome analysis in almond, we used the cold tolerant genotype H that is an indigenous genotype to Iran. The genotype H is grown in the Kamal-Shahr Collection Orchard, Seed and Plant Improvement Institute (SPII), 50 Km west of Tehran, Iran. The geographical specifications of the region are as follow: latitude 35.55 N, longitude 50.54 E and altitude 1312.5 M above the sea level. Natural spring frost of −3°C (±0.5°C) has been recorded in this region annually. The H genotype was identified by breeders as one of the cold-tolerant genotype among 300 genotypes, collected from all over Iran and grown in the same region. This genotype is capable of resisting freezing temperatures in the field as shown during five years of evaluation (unpublished data) and is set to be released as a cold resistant cultivar. Because the pistil is the critical organ for production of nut, brownish pistils were considered as phenotypic marker in the evaluation of frost damages [22]. Additionally, a preliminary study showed that genotype H had physiological characteristics of cold tolerance when compared to other genotypes [57]. The H genotype is a late blooming genotype. At −2°C (±0.5°C) only 5% of the flowers were severely damaged, therefore it allowed us to collect samples to study gene expression. In the popcorn stage (Figure 7A), six branches were collected from the top to middle of five-year old H genotype trees during early spring (March 2013). The diameter and the length of the branches were approximately 0.5 cm and 35 cm, respectively. The cut-ends of the branches were held in a 5% sucrose solution during transport to the laboratory and the frost treatment was immediately applied. The cut branches were subjected to freezing temperature (−2°C) for 5 h (programmable freezing chamber) and ovary and anther samples (Figure 7B) were collected from 8–10 flowers. The mentioned plant stage (popcorn) and frost treatment (−2°C) were selected according to Imani et al.’s [21], [22] findings. As control treatment, several cut branches were kept at 10°C. The collected samples were immediately frozen in liquid nitrogen and stored at −80°C.

### RNA Isolation and Sequencing

Total RNA was isolated from anther and ovary samples using previously discribed method of Yang et al. [58] with the following modifications. Briefly, 1 ml of extraction buffer (2% W/V CTAB; 0.1 M Tris-HCl (pH 8); 1.4 M NaCl; 20 mM EDTA (pH 8); 2% (w/v) PVP; 70 µl of β-mercaptoethanol) was added to ∼100 mg fine powder of each sample and incubated at 65°C for 10 min. Then 800 µl chloroform were added to the mixture and centrifuged at 10,000 rpm for 10 min at 4°C. Next, 800 µl phenol/chloroform (1∶1 V/V) were added to the supernatant and centrifuged once more (10,000 rpm for 10 min at 4°C). An equal volume of cold chloroform/isoamyl alcohol (24∶1) was added to the supernatant and centrifuged (10,000 rpm for 10 min at 4°C). Finally, following the addition of LiCl (8 M) and centrifugtion, the pellets were washed with ethanol and dissolved.

The RNA preparations were treated with RNAse-Free DNaseI (Promega) following manufacturer’s protocol. In brief, 15 µl of DNaseI buffer and 4 µl DNase-Free RNase were added and the mixture was incubated for 30 min at 37°C. Later 15 µl EDTA (50 mM) was added and incubated for 10 min at 65°C. Following extraction by phenol/chloroform and chloroform/isoamyl alcohol, total RNA content of samples were precipitated by sodium acetate (3 M) and ethanol 100%, and incubated at −20°C for 3 h. The RNA was recovered by centrifugation (13,000 rpm for 15 min at 4°C) and washed with 70% ethanol.

The quantity and quality of RNA in each sample were estimated using a spectrophotometer and agarose gel electrophoresis. Samples with higher quality were selected for sequencing. The cDNA library construction and sequencing procedure (on a Illumina HiSeq 2000 platform) were perfomed by Macrogen company (Korea). Four paired-end sequencing libraries with insert sizes of about 101 bp were constructed, which resulted in a total of 24,451,191 reads. RNA-Seq data were produced in FASTQ format, which was obtained from the company’s server. The raw transcriptome data has been deposited in the sequence reads archive (SRA), NCBI, and could be accessed using SRP041269 accession number.

### *De Novo* Transcriptome Assembly and Homology Search

The quality assessment software FastQC was used to assess the quality of the reads (http://www.bioinformatics.babraham.ac.uk/projects/fastqc/). Reads with quality lower than Q30 were filtered out and the remaining reads were trimmed (15 nt from 5′ end) using the FastX-toolkit software suite (http://hannonlab.cshl.edu/fastx_toolkit/). All post-processed reads from the four libraries were assembled *de* *novo* using Trinity v1.3 [59] with k-mer sizes set at 25 using a PC running Linux OS (Ubuntu 12.04 LTS) on 8 CPUs (2.6 GHz) and 16 GB of RAM. Assembly of each library was typically completed within 24 hours. *De novo* transcripts were annotated using BLASTX (version 2.2.25) [60], with a cut-off Evalue of 10^−5^. For each transcript, the best BLAST hit was selected for the following analysis.

### Validation of *De Novo* Assembly

To verify the accuracy of *de* *novo* assembly and annotation, we mapped reads on our assemblies and the peach (*Prunus persica*) genome v1.0 [11]. Mapping statistics were obtained using Bowtie v0.12.8 package, following the default parameters [24].

### Gene Expression Profile

Transcript quantification for RNA-seq reads was performed with RSEM based on mapping the RNA-seq reads to the assembled transcriptome [61]. RSEM estimated fraction of transcripts (as value between zero and 1 or can be multiplied by 10), which were further used to obtain mesurements in terms of transcript per milliun (TPM).

Differential gene expression analysis was performed using EBseq [62] within RSEM to compare control and frost treated libraries. EBSeq is an empirical Bayesian approach that models a number of features observed in RNA-seq data.

### Quantitative Real Time PCR

To validate the results of RNA-seq analysis, a new experiment was conducted during spring (March 2014). The procedures of frost treatment, sampling and total RNA extraction from anther and ovary tissues were the same as described above. Approximately, 1 µg of total RNA per sample was subjected to reverse transcription using reverse transcription system (Qiagene). The qRT-PCR was performed with SYBR Premix *Ex Taq* (Takara) on a Roter-Gene Q (Qiagen). Each reaction contained 1 µL of the first-strand cDNA as template, in a total volume of 12 µL reaction mixture. The amplification program was performed as 95°C 30 s followed by 95°C for 5 s and 60°C for 30 s (40 cycles). The list of gene-specific primers were shown in Table 3. The qRT-PCR expression levels were analyzed based on the mean of two biological and three technical repeats. Relative gene expression level was obtained using the 2^−ΔΔCT^ method [63]. *PdAct1* gene was used as an internal control for normalization of data [64].

### Gene Ontology Analysis

Gene set enrichment analysis was performed on the resulting lists of significant DE genes using AgriGO analysis tools which were accessed via http://bioinfo.cau.edu.cn/agriGO. Fisher’s exact test (*p*-value<0.05) was used to compare the DE list with peach genome. In addition, Greller and Tobin, R of Stekel and Falcianiand General Chi-squared tests were all conducted for comparison of GO list among DE lists and tissues using IDEG6 web tool [37].

## Supporting Information

File S1 **Contains the files: Table S1.** The expression profiles of genes identified in anther tissue under control (HCA) and frost stress (HSA). The complete list of expressed genes and their level of expressions given in transcript per million (TPM). TPM_HSA and TPM_HCA indicate expression of gene under stress and control conditions, respectively. **Table S2.** The expression profiles of genes identified in ovary tissue under control (HCO) and frost stress (HSO). The complete list of expressed genes and their level of expressions given in transcript per million (TPM). TPM_HSO and TPM_HCO indicate expression of gene under stress and control conditions, respectively. **Table S3.** Gene expression profiles of commonly occuring transcript that are present in both ovary and anther tissues. The complete list of expressed genes and their level of expressions given in transcript per million (TPM). TPM_HSO and TPM_HSA indicate expression of gene under stress condition in ovary and anther, respectively. While, TPM_HCO and TPM_HCA show expression under control conditions. **Table S4.** Differentially expressed genes in anther tissue of almond under frost (HSA *vs.* HCA). Differentially expressed genes were identified using EBSeq package. The expression of any individual transcript was obtained by comparing its expression under stress condition (HSA) to that of control condition (HCA). The fold change cut-off was set at two fold with *p*-value<0.05. **Table S5.** Differentially expressed genes in ovary tissue of almond under frost (HSO *vs.* HCO). Differentially expressed genes were identified using EBSeq package. The expression of any individual transcript was obtained by comparing its expression under stress condition (HSO) to that of control condition (HCO). The fold change cut-off was set at two fold with *p-*value<0.05. **Table S6.** Biological process of differentially expressed genes in almond under frost stress. The complete list of biological processes is presented for the DE genes in both ovary (HSO *vs.* HCO) and anther (HSA *vs.* HCA). **Table S7.** Molecular function of differentially expressed genes in almond under frost stress. The complete list of Molecular functions is presented for the DE genes in both ovary (HSO *vs.* HCO) and anther (HSA *vs.* HCA). **Table S8.** Cellular comportments of differentially expressed genes in almond under frost stress. The complete list of cellular compartments is presented for the DE genes in both ovary (HSO *vs.* HCO) and anther (HSA *vs.* HCA). **Table S9.** Comparative analysis of Gene ontology of over-expressed genes in anther between almond and peach reference. Fisher’s exact test with *p*-value<0.05 was conducted to comparison of GO terms. **Table S10.** Comparative analysis of Gene ontology of down-expressed genes in anther between almond and peach reference. Fisher’s exact test with *p*-value<0.05 was conducted to comparison of GO terms. **Table S11.** Comparative analysis of Gene ontology of over-expressed genes in ovary between almond and peach reference. Fisher’s exact test with *p*-value<0.05 was conducted to comparison of GO terms. **Table S12.** Comparative analysis of Gene ontology of down-expressed genes in ovary between almond and peach reference. Fisher’s exact test with *p*-value<0.05 was conducted to comparison of GO terms.
(XLSX)
